# Author’s Response to Peer Reviews of “Converting Organic Municipal Solid Waste Into Volatile Fatty Acids and Biogas: Experimental Pilot and Batch Studies With Statistical Analysis”

**DOI:** 10.2196/69894

**Published:** 2025-02-04

**Authors:** Hojjat Borhany

**Affiliations:** 1 Faculty of Environmental Science, Department of Environmental Science, Informatic, and Statistics, University of Ca' Foscari Venice, Mestre (VE), Italy

**Keywords:** multistep fermentation, specific methane production, anaerobic digestion, kinetics study, biochar, first-order, modified Gompertz, mass balance, waste management, environment sustainability

*This is the author’s response to peer-review reports of “Converting Organic Municipal Solid Waste Into Volatile Fatty Acids and Biogas: Experimental Pilot and Batch Studies With Statistical Analysis.”*

## Round 1 Review

### Anonymous [1]

*The present manuscript [* *2* *] deals with the study of the valorization of organic fractions of municipal solid waste through the production of volatile fatty acids (VFAs) and biogas. The article is interesting; in my opinion, it should be revised.*

#### Comments

*1. The presentation of the manuscript is very poor; the figures are not in the same format.*

**Response:** The remaining figures, which included the box plots of VFA concentration, VFA/soluble chemical oxygen demand (SCOD) ratio, scheme of line, VFA and SCOD concentration, VFA weight ratio distribution, capital cost and yearly income, and biomethane content, were kept and reformulated to have the same shape. The figures outlining the kinetics study were deleted.

*2. Some of the recent works should be discussed and cited in the Introduction section: [3-7].*

**Response:** Some of the recent relevant works and studies were discussed and cited in the Introduction section as follows:

- Inyang M, Gao B, Pullammanappallil P, Ding W, Zimmerman AR. Biochar from anaerobically digested sugarcane bagasse. *Bioresour Technol*. Nov 2010;101(22):8868-8872. [doi: 10.1016/j.biortech.2010.06.088] [Medline: 20634061]
- Jung S, Shetti NP, Reddy KR, et al. Synthesis of different biofuels from livestock waste materials and their potential as sustainable feedstocks – a review. *Energy Conversion Manage*. May 15, 2021;236:114038. [doi: 10.1016/j.enconman. 2021.114038]
- Sampath P, Brijesh, Reddy KR, et al. Biohydrogen production from organic waste – a review. *Chem Eng Technol*. Jul 2020;43(7):1240-1248. [doi: 10.1002/ceat.201900400]
- Algahashm S, Qian S, Hua Y, et al. Properties of biochar from anaerobically digested food waste and its potential use in phosphorus recovery and soil amendment. *Sustainability*. Dec 10, 2018;10(12):4692. [doi:10.3390/su10124692]

*3. The novelty of the work should be highlighted.*

**Response:** We noted at the end of the Introduction and at the beginning of the Discussion that this study is novel in that it presents a strong framework for evaluating a proposal for the financial and technical valorization of organic municipal solid waste using statistical analysis, process kinetics, mass balance, and experimental testing. Furthermore, as compared to single-step anaerobic digestion, our data showed a notably high improvement in profitability and a corresponding decrease in the payback period. In order to further close the cycle circuit and prolong the product life, we also proposed the integration of two potential future units.

*4. Full stops should be removed from all subheadings.*

**Response:** They are all removed.

*5. The Results and Discussion should be written in detail with proper subheadings.*

**Response:** The Results section was rewritten and divided into subheadings to mirror their counterparts in the Methods, and the Discussion section has the added subheadings Principal Results, Comparison With Previous Works, and Conclusion and Limitations according to the required information in the guidelines of JMIR Publications.

*6. There are some typo errors; they should be rectified.*

**Response:** They were corrected.

### Reviewer GA [8]

#### General Comments

*Generally, the manuscript should be strictly improved in English language writing and corrected for all grammatical errors throughout the whole manuscript. The author has to use a uniform style of the English language, either American or British English. Further English assistance is particularly required. Many missing articles and a lot of grammatical and punctuation errors must be corrected in the manuscript as in the corrected abstract.*

**Response:** The abstract was prepared in an organized format and corrected for its language. We also employed English assistance. The manuscript’s English was improved, and its style was harmonized with American English.

#### Specific Comments

*This paper shows an important aspect of multiple fermentation steps for the complete utilization of municipal solid waste and conversion to useful products, which is highly recommended for circular economic sustainability worldwide. However, it needs some major revision and arrangement to allow for a better presentation of this valuable work.*

##### Major Comments

###### Title

*1. “Valorization of Organic Fraction of Municipal Solid Waste Through Production of Volatile Fatty Acids (VFAs) and Biogas” is a long title that should be shortened to be more concise with no abbreviations—more indicative. Suggested title: “Valorization of Organic Municipal Solid Waste for Volatile Fatty Acids and Biogas Production.”*

**Response:** It was adopted according to the guidelines for the descriptive title of the original paper: “Issue/Intervention in Demographic/Disease/Condition: Method/Study Design”; “Conversion of Organic Municipal Solid Waste to Volatile Fatty Acids and Biogas: Experimental Pilot and Batch Studies with Statistical Analysis.”

###### Abstract Section

*2. General language; it must be more concise and specific.*

**Response:** I did search for all the general language in the manuscript and tried to provide concise information on the matter.

*3. Please clearly mention the take-home message and the main findings of the research.*

**Response:** The research’s primary conclusions include the development of a reliable technique for evaluating the recovery proposal for the conversion of organic solid waste into valuable products and assessing both its technical and financial viability. Furthermore, our proposal outperforms the conventional approaches in terms of economics.

*4. The abstract is too long and lacks the main methodology and main experimental techniques that were carried out in this work. The author may add some hints about the main methods used before mentioning the main results.*

**Response:** Subheadings for the background, objective, method, findings, and conclusion were added to the revised abstract. There are fewer words in the abstract overall than the 450-word limit. Additionally, some pointers regarding experimental techniques such as gas chromatography are provided, along with the kind of statistical test used to verify the significance and efficacy of the suggested process amendment. We also mentioned the use of mass flow models for the process’s economic evaluation and the various kinetics models that can be used to describe biogas production.

###### Manuscript

*5. Keywords: Words must be modified to be more informative and representative of the research interest and differ from the word in the manuscript title. Maybe add “Multi Step of Fermentation Process” or “Waste Management and Environment Sustainability.”*

**Response:** We updated the keywords to include “Multistep Fermentation,” “Environment Sustainability,” “Waste management,” “Specific Methane Production,” “Anaerobic Digestion,” “Kinetics Study,” “Biochar,” “First-Order,” “Modified Gompertz,” and “Mass Balance.”

*6. Arrangement of the experimental work in the manuscript may be needed in the Results and Discussion accordingly.*

**Response:** It was completed in a way that would make it easier for specialists in the field to follow the stages, and a Discussion section was included to compare the findings with earlier research, highlight the key conclusions, and clarify the research’s limitations.

*7. There is a lack of figures to describe the main parameter optimization steps well. Please reformulate to describe some data using figures with error bars.*

**Response:** Our optimization procedure focused on reducing the payback period by decreasing the cost and increasing the profit from bioproducts. This was achieved through pilot tests for examining the effectiveness of the hydraulic retention time (HRT) manipulation and pretreatment in increasing the VFA yield and the integration of our process knowledge of using the fine-tuned feedstock/inoculum ratio as well as biochar addition to obtain the biogas in a cost-effective process. Detailed information and calculations regarding the mass flow analysis are available in the supplementary documents in the Excel spreadsheet named“Mass Balance.”. For figures, we provided the VFA concentrations and distribution for two HRTs and a *t* test to confirm the significance of the results. Further, for biogas production, we provide results from a kinetics study showing an 8-fold increase in the hydrolysis rate and a 100% decrease in the lag phase. This brought about a small anaerobic digester working at a high organic loading rate, leading to a reasonably priced process.

*8. The SD and table footnotes with the number of replicates should be noted underneath all of the given tables.*

**Response:** For all data that was accompanied by an SD, the number of replicates was reported beneath all the given tables.

*9. A mechanistic in-detail discussion is required, not just comparing your results with the previous work; justify better.*

**Response:** The comparisons of results from similar studies were done mechanistically and in detail.

For example:

- “Because of the extra pretreatment unit in our study, our VFA yield was significantly higher than the study by valentino et al ”
- “The higher hydrolysis rate was due to the destruction of the solids structure caused by bacterial enzymes and a hot alkaline solution. Additionally, we provided a higher active biomass per feedstock using a fine-tuned FS/IN ratio of 0.3 (VS basis), which was noticeably lower than the quantities (1 and 0.5) reported in similar studies ”
- “due to the added fresh WS with higher digestible content and better nutrient balance than the fermented solids, the SMP value by valentino et al was higher.”
- “The higher practicability than the 2 steps of bioethanol and biogas production as a result of sterilization and high bioethanol concentration requirements.”
- “Our proposal is more favorable since it does not limit the VFA weight ratio distribution and does shifts the recovery route toward higher market-valued products like VFAs than single step AF + AD by Papa et al”

*10. In research articles, do not include any table comparing literature results; the author can discuss the main findings in the text itself, as in Table 5.*

**Response:** All the data in the tables comparing results were deleted, and we discussed them in the text.

*11. The Conclusions section is missing in the manuscript to summarize and point out the novelty and the main findings from the research.*

**Response:** The Conclusion was included in the manuscript and presents the main findings as follows: “To conclude, we presented a robust framework to assess a proposal for the valorization of organic waste through experimental tests, statistical analysis, and process kinetics, along with mass and energy flow analysis. The findings support considerably higher profitability and, as a result, a shorter payback period for multistep reclamation than the current single anaerobic digestion. Further, our results encourage the circular economy perspective on the conversion of OMSW into biogas and VFAs, with the pros of fewer residual solids due to reusing them in a pyrolysis line.”

*12. Generally speaking, in academic writing, (1) abstracts do not include abbreviations, (2) avoid articles in the title (the, a, an), and (3) avoid keywords that exist in the title.*

**Response:** (1) Based on JMIR House Style and Guidelines, the usage of abbreviations and acronyms in the abstract section is not forbidden. Further, all author-invented abbreviations were omitted. We also stop using “AD” as an abbreviation for anaerobic digestion since it may make it ambiguous with “AD” (the reference year). In fact, keeping the number of words in the abstract within the limits is really impossible without using some of them. (2) It was avoided. (3) It was avoided to be as informative as possible.

*13. As a rule of thumb, no dots in titles or subtitles as in the Experimental section, Anerobic Pilot Unities, etc.*

**Response:** The dots were removed.

*14. Multiple references should be merged, not written separately, as in “29, 30” and “23, 27”; the author may use the merge reference option in reference software.*

**Response:** It was corrected.

*15. The author may add numbers for all titles and subtitles accordingly all over the manuscript.*

**Response:** Based on the JMIR guidelines for the author, it is not allowed to use numbering for headings and subheadings.

### Minor Comments

*16. The author should avoid general and well-known information, and be selective in the recent references used. May add one small paragraph to the Biological Waste Management and Environment Sustainability section.*

**Response:** The small paragraph already discussed the current state of municipal organic waste production and treatment in the European Union. We extended it and incorporated all other information regarding environmental sustainability from some relevant sources suggested by the peer reviewer.

*17. The author should clarify the main aim of the work clearly in the last paragraph of the Introduction.*

**Response:** The main aim of this study was an assessment of multistep pretreatment acidogenic fermentation, followed by anaerobic digestion of municipal organic waste in comparison with the existing method of single anaerobic digestion in terms of financial profit and technical feasibility.

*18. Do not use our, we, or us in academic writing.*

**Response:** Based on the journal guidelines, there are no issues with using we and us in the article submitted to JMIR Publications; nevertheless, I do my best to avoid overusing these words in my manuscripts.

*19. The author may mention novel applications of VFA and biogas. Mention different novel sources of biogas production.*

**Response:** It was already mentioned in the study that biogas and VFA typically were used for energy production and biopolymer synthesis, respectively. Moreover, other sources of biogas typically were from nonbiological processes, which were beyond our scope since we focused on carbon-neutral microbiological processes.

*20. The author should mention the gas chromatography type, gas injection rate, column dimensions, and the used carrier gas in the main document.*

**Response:** It was included in the Methods section.

*21. The author did not mention that flushing with nitrogen or carbon dioxide took place in anaerobic digestion while feeding reactors and how the anaerobic conditions were maintained; please mention it clearly or add the references used for the methodology.*

**Response:** The anaerobic condition was ensured in bottles just by sealing them after filling without any flushing with nitrogen or carbon dioxide since we had known that the oxygen transfer at the surface of the waste stream was impossible as it contained high total solids and SCOD. This type of procedure was adopted in our lab and has been conducted for years.

*22. Organize titles all over the manuscript.*

*23. Generally, the subtitles are too generic; modify them to be more indicative and precise.*

**Response:** The subtitles were modified to be more indicative and precise.

*24. “unless Saturday and Sunday” in line 208 is not important information; the suggested word “daily” is enough.*

**Response:** It was corrected.

*25. “Unite”: Please correct.*

**Response:** All units are corrected.

*26. Remove the grid lines in the figures.*

**Response:** They were removed.

*27. The author has to mention the range used for the chemical oxygen demand method, and the original reference should be cited appropriately.*

**Response:** The method for determination of soluble and solid chemical oxygen demand of the waste stream was according to the Standard Methods for Water and Wastewater. We also clearly discussed in the Methods section a proper limit of detection and reference.

*28. “As can be seen”: This statement is repetitive more than once in the Discussion, in lines 301, 315, and 423.*

**Response:** Line 301 was corrected. Line 315 was corrected to be informative and avoid repetition. Line 423 was rectified in English language, and the repetitive statements were removed.

*29. Figure 3 caption: Mesophilic fermentation: Please specify which stage because both of the sequential steps were called mesophilic fermentation in Figure 1.*

**Response:** In fact, Figure 3 depicts the weight ratio distribution from the second step named mesophilic acidogenic fermentation. Surprisingly, the VFA could only be obtained from the second stage. Additionally, we modified the caption to read “VFAs weight ratio distribution for mesophilic acidogenic fermentation” and made a clear reference to Figure 1, which depicts the processes of pretreatment, acidogenic fermentation followed by mesophilic anaerobic digestion . In terms of pH and HRT, the two later procedures differ from one another substantially.

*30. What is the rationale for comparing 3 days to 4.5 days for all the used systems; the author may justify why 4.5 days is better to complete with this HRT in the rest of the experiments or describe the one variable at a time optimization method that is used to determine the significant factors and the insignificant one; mention them clearly. Also, use in the Discussion the terms “significant” and “insignificant” according to the obtained P value.*

**Response:** The values for the two HRTs to increase the VFA concentration in the outlet were selected based on our experience and process knowledge. According to this information, exceeding the HRT value of more than 3‐5 days can bring the process into an anaerobic digestion step. As a result, the VFAs with high-added value markets are converted to biogas. Hence, the two HRTs of 3 days and 4.5 days were tried in the pilot test, knowing that the VFA concentration would either increase or decrease linearly in this local region of operation.

*31. The author has to mention tables and figures in the text in their appropriate place.*

**Response:** They were mentioned where they were referred to.

## Abbreviations

HRT: hydraulic retention time
SCOD: soluble chemical oxygen demand
VFA: volatile fatty acid
